# RAMP1 as a novel prognostic biomarker in pan-cancer and osteosarcoma

**DOI:** 10.1371/journal.pone.0292452

**Published:** 2023-10-05

**Authors:** Long Xie, Weiwei Xiao, Hangqi Fang, Guoqiang Liu

**Affiliations:** 1 Trauma Department of Orthopaedics, The Affiliated Yuebei People’s Hospital of Shantou University Medical College, Shaoguan, Guangdong Province, China; 2 Gastroenterology Department, The Affiliated Yuebei People’s Hospital of Shantou University Medical College, Shaoguan, Guangdong Province, China; Universite de Nantes, FRANCE

## Abstract

Receptor activity modifying protein 1 (RAMP1) facilitates the localization of the calcitonin-like receptor (CLR) to the plasma membrane, but its role in osteosarcoma (OS) remains unclear. We evaluated the RAMP1 expression and prognostic value across different cancers, studying tumor immune infiltration. The prognostic value was analyzed using the GSE39058 and TARGET datasets. Differential gene expression was evaluated. a protein-protein interaction network was constructed, and gene set enrichment analysis was performed. The function of RAMP1 in the tumor microenvironment was analyzed, and its expression in OS cell lines was validated using quantitative real-time PCR. High RAMP1 expression correlated with poor prognosis relative to low RAMP1 expression (*p* < 0.05). Low RAMP1 expression correlated with an abundance of CD4+ memory-activated T cells. whereas a high expression level correlated with a high proportion of gamma-delta T cells (γδ T cells). Differentially expressed genes from TARGET was enriched in olfactory transduction pathways (normalized enrichment scores [NES] = 1.6998, *p* < 0.0001). RAMP1 expression negatively correlated with CD44 expression but positively correlated with TNFSF9 expression. The RAMP1 gene is substantially expressed in OS cells compared to the normal osteoblast cell line hFOB1.19. Thus, RAMP1 may be a prognostic biomarker and potential therapeutic target in OS.

## Introduction

Osteosarcoma (OS), the most prevalent kind of primary malignant bone tumor, typically affects children, teenagers, and young adults. Nearly one-fourth of patients with OS present with metastatic disease, with the lungs being the most common site [1, 2]. Despite recent breakthroughs in both chemotherapy using multiple agents, and surgical local control as the primary treatment for OS, patients with lung metastases have an extremely poor prognosis due to the challenge of controlling and/or alleviating multifocal disease over an extended period [3, 4]. Consequently, there is an urgent need for novel predictive biomarkers to support clinicians in determining more effective treatment strategies, particularly immune-based therapies, and to assist in the planning of future clinical trials.

The receptor activity modifying protein (RAMP) family consists of three proteins: RAMP1, RAMP2, and RAMP3, all of which exhibit<30% sequence homology and a comparable structure [5]. RAMP1 is increasingly acknowledged to have a crucial role in the progression of non-neoplastic disorders in addition to the genesis and progression of cancer. Previous studies have indicated that DNA methylation at the RAMP1 promoter may play a role in migraines, with a low level of methylation at CpG units potentially serving as a risk factor for females [6]. Moreover, RAMP1 overexpression promotes mouse skin fibroblast (MSF) cell proliferation via the Gi3-PKA-CREB-YAP axis, suggesting that RAMP1 function is a new target for the treatment of skin wounds [7]. In the field of tumor research, RAMP1 has been identified as a direct NKX3.1 target gene and a novel biomarker, playing a significant role in promoting prostate carcinogenesis. Consequently, RAMP1 is crucial for the development of innovative therapies for prostate cancer [8]. Furthermore, RAMP1 knockdown has been demonstrated to decrease the clonogenic growth rate and tumorigenic potential of Ewing sarcoma (EwS) cell lines, indicating a novel therapeutic approach for inhibiting EwS progression [9]. However, the function of RAMP1 in OS metastasis and its clinical significance remain to be elucidated.

In this study, the bioinformatic analysis demonstrated that the level of RAMP1 expression was related to a metastatic phenotype and a poor prognosis. High RAMP1 expression was related to poor overall survival and distant metastases in patients with OS, Additionally, we conducted in vitro experiments to investigate the changes in RAMP1 gene expression between the hFOB1.19 osteoblast cell line and the OS cell line. Collectively, these finding suggest that RAMP1 holds promise as a potential molecular target for OS treatment and as a potential biomarker for the diagnosis and prognosis of OS.

## Material and methods

### Expression and diagnostic value of RAMP1 in pan-cancer

The Cancer Genome Atlas (TCGA) (https://portal.gdc.cancer.gov) was mined for pan-cancer analysis data, RNA-sequencing expression profiles, and therapeutically relevant information. The relationship between RAMP1 expression and overall survival, disease-free survival (DFS), and progression-free survival (PFI) was analyzed using Kaplan-Meier plots. To perform this analysis, we used the "survminer" and "survival" packages. Statistical significance was defined as *p* < 0.05.

### Relationship between RAMP1 and immune cells in the tumor microenvironment (TME) in pan-cancer

To investigate potential relationships between RAMP1 and the TME, we performed a TCGA pan-cancer analysis and generally explored the effect of RAMP1 on immune infiltration in the TME) using an online database, Sangerbox [10].

### Analysis of differentially expressed genes (DEGs) in the GSE39058 dataset

We downloaded normalized gene-level RNA-seq and clinical data for GSE39058 [11] from the Gene Expression Omnibus (GEO) database (https://www.ncbi.nlm.nih.gov/geo). DEGs were obtained from the expression profile dataset GSE39058 using the criteria *p* < 0.05 and |logFC| > 0. To confirm the role of the RAMP1 gene in the development of OS, data from the Therapeutically Applicable Research to Generate Effective Treatments (TARGET) (https://ocg.cancer.gov/programs/target) were obtained and examined.

### Gene Ontology (GO) analysis in the GSE39058 dataset

In this study, multiple online databases and R packages were used to analyze candidate DEG functions and enrichment pathways. DEGs were screened using the "limma" package of the "R" software [12], and heat map was constructed using the "pheatmap" package. For statistical analysis, the "survival" and "survminer" package were employed. A user-friendly website, GeneMANIA [13] (http://genemania.org), provided a research platform for creating hypotheses on gene function, analyzing gene lists, and ranking genes for functional tests. Additionally, the Gene Set Enrichment Analysis (GSEA) [14] was used to investigate the relationship between gene sets and biological signals within a single dataset.

### Potential role of the RAMP1 gene in the prognosis of OS in the TARGET dataset

The prognostic value of the RAMP1 gene was evaluated using receiver operating characteristic (ROC) analysis and is represented by the area under the ROC curve (AUC) score. According to the usual ranking, 0.9 ≤ AUC ≤ 1 is regarded as outstanding, 0.80 ≤ AUC <0.9 as good, 0.70 ≤ AUC < 0.8 as adequate, 0.50 ≤ AUC < 0.7 as inadequate, and AUC < 0.5 as a failure. A nomogram model was constructed using the R packages "regplot", "timeROC", and "rms". Calibration plots were used to verify the model’s calibration. Additionally, an overall survival forest plot was constructed using combined hazard ratios (HR) (95% confidence interval [CI]) based on both univariate and multivariate Cox regression analyses.

### Functional enrichment and GSEA analysis of DEGs in the TARGET dataset

A circular visualization plot was constructed using the "circlize" R package [15]. Kyoto Encyclopedia of Genes and Genomes (KEGG) analyses were performed using the "ggplot2" package for visualization and the "Cluster Profiler" package [16] for statistical analysis. The GSEA analysis was performed using the gene set collections C2: CP-KEGG (KEGG gene sets) and C5 (GO gene sets) from Molecular Signature Database (MSigDB) v6.0 [17].

### Correlation analysis of RAMP1 in the TARGET dataset

The correlation analyses of RAMP1 gene expression were conducted using Pearson’s correlation coefficients and a two-tailed test. Values of ≥0.5 for Pearson’s coefficient and <0.05 for the *p*-value indicated a substantial positive association.

### Association between immune infiltrating level and RAMP1 expression in OS

To describe the immune and stromal microenvironment of OS, CIBERSORT [18] was utilized to evaluate the infiltrating abundance of 22 immune cells between the high and low RAMP1 expression groups. Additionally, immune and stromal scores were calculated using ESTIMATE [19]. The association between RNA expression of immune checkpoint-related genes and the RAMP1 gene was analyzed and visualized. In addition, Spearman’s rank test was used to analyze the association between RAMP1 expression and possible gene markers of tumor-infiltrating immune cells.

### Cell culture

The MG63 and HOS human OS cell lines were obtained from the American Type Culture Collection (ATCC, USA). The cells were cultured in Dulbecco’s modified Eagle’s medium (DMEM) (Gibco, USA) supplemented with 10% fetal bovine serum (FBS) (Excell, China). All cells were incubated at 37°C in a humidified atmosphere with 5% CO2. After five days of infection, total RNA was extracted from the cells using TRIzol reagent (Invitrogen) according to the manufacturer’s instructions. Next, cDNA was synthesized from the total RNA. Reverse transcription was performed using M-MLV reverse transcriptase (Promega, Madison, WI, USA). Quantitative polymerase chain reaction (qPCR) was employed in determining the expression level of RAMP1 using an SYBR-Green Master Mix (Takara Biotechnology Co., Ltd.). β-actin was used as an internal control. Relative gene expression levels were calculated using the 2^- ΔΔ Ct^ method. Two sets of primers were used for the PCR amplification-β-actin forward primer: 5’-CACCATTGGCAATGAGCGGTTC-3’ and reverse primer: 5’-AGGTCTTTGCGGATGTCCACGT-3’; RAMP1 forward primer: 5’-TAACTACGGTGCCCTCCTCC-3’ and reverse primer: 5’-GGCCAGCTCCCTGTAGCTC-3’.

### Statistical analysis

Each experiment was performed in triplicate. All statistical analysis was performed using GraphPad Prism 6.0. Data are presented as mean ± standard deviation. The threshold for statistical significance was set to *p* < 0.05.

## Results

### RAMP1 gene expression in different tumor types and prognostic implications of RAMP1 expression

**Fig 1:**
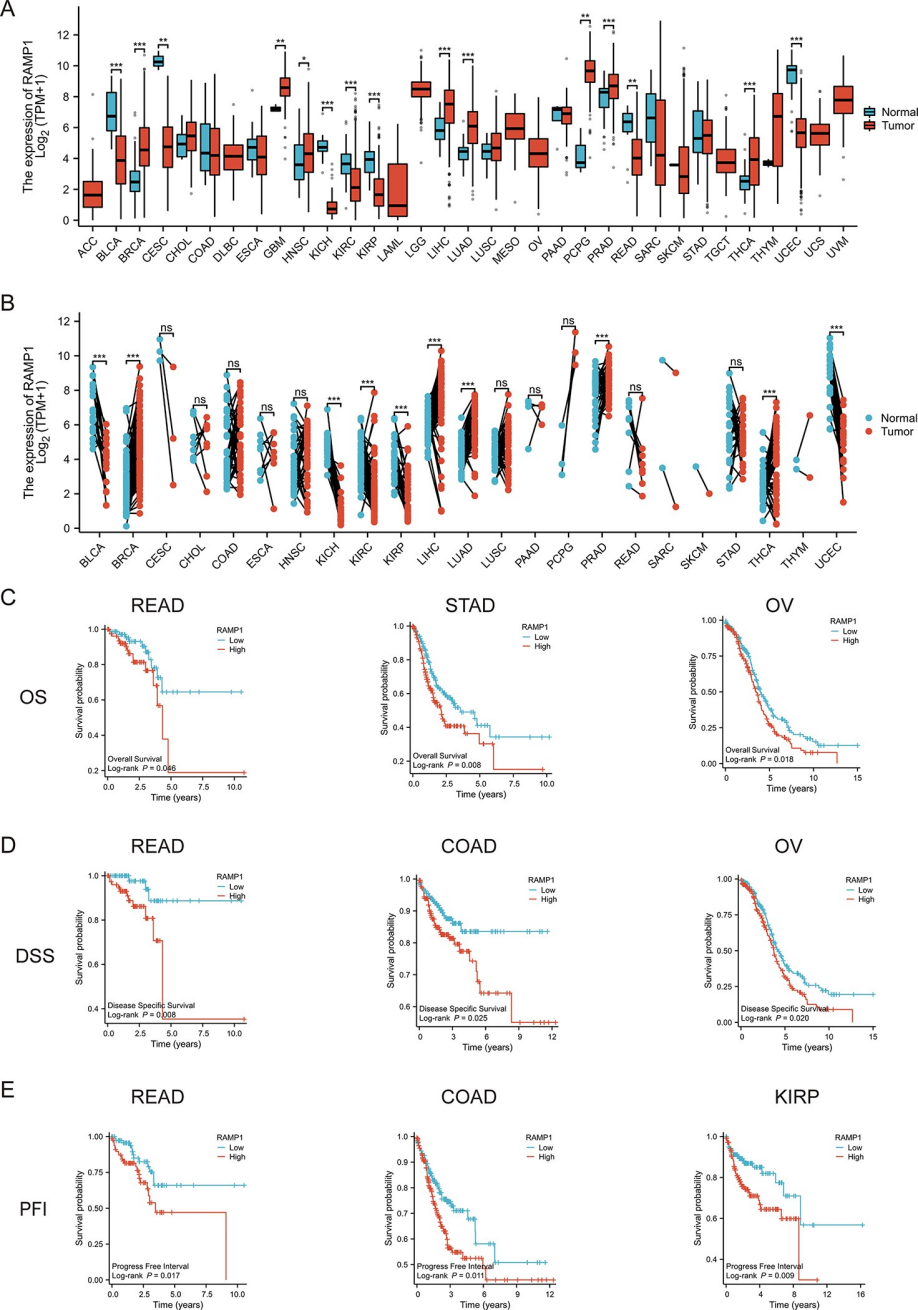
Receptor activity modifying protein 1 (RAMP1) expression and prognostic implications in pan-cancer. There was an upregulation of RAMP1 in breast invasive carcinoma (BRCA), prostate adenocarcinoma (PRAD) and liver hepatocellular carcinoma (LIHC). Conversely, downregulation of RAMP1 was observed in bladder urothelial carcinoma (BLCA) and uterine corpus endometrial carcinoma (UCEC) (1A and 1B). High RAMP1 expression levels were associated with poor clinical outcomes in rectal adenocarcinoma (READ) (1C-1E).

Several studies have investigated the expression levels of the RAMP1 gene across various tumor types. For instance, research has demonstrated the upregulation of RAMP1 in breast invasive carcinoma (BRCA), prostate adenocarcinoma (PRAD), and liver hepatocellular carcinoma (LIHC). Conversely, downregulation of RAMP1 has been observed in bladder urothelial carcinoma (BLCA) and uterine corpus endometrial carcinoma (UCEC) (Fig 1A and 1B). The altered expression of RAMP1 has shown significant associations with cancer prognosis. High RAMP1 expression levels have consistently been correlated with unfavorable clinical outcomes in rectal adenocarcinoma (READ), including shorter overall survival and decreased DFI and PFI (Fig 1C–1E).

### Correlation of RAMP1 gene with immune checkpoints, immune cell analysis, and immune scores

**Fig 2:**
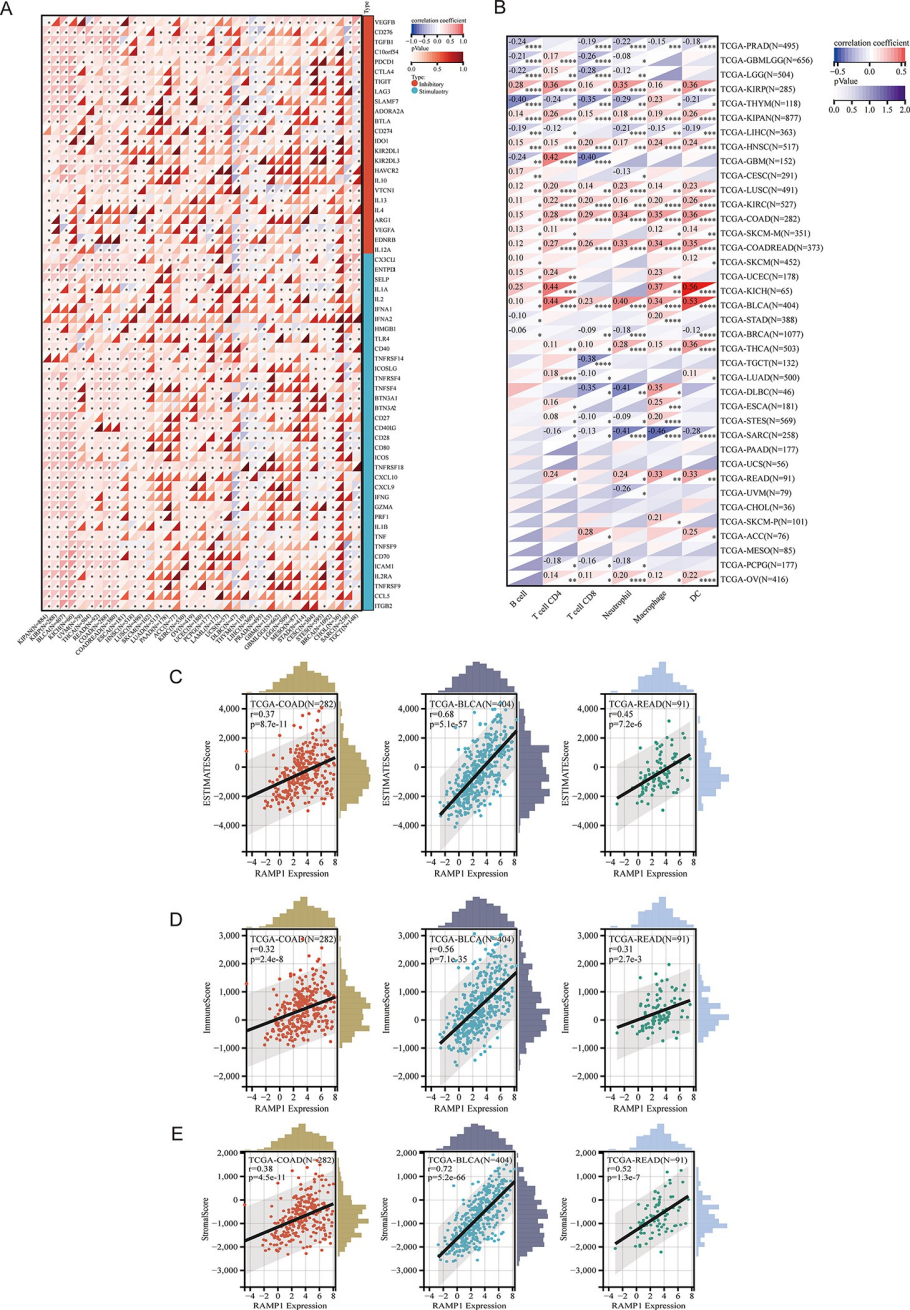
Correlation between RAMP1 gene expression and immune checkpoint molecules in pan-cancer studies (2A); various immune cell populations infiltrating the tumor microenvironment (2B); and immune scores, including ESTIMATE Score, Immune Score, and Stromal Score (2C-2E).

The analysis revealed a significant correlation between RAMP1 gene expression and immune checkpoint molecules. Increased RAMP1 expression was strongly associated with the upregulation of lymphocyte activation gene 3 (LAG3), vascular endothelial growth factor B (VEGFB), and cytotoxic T-lymphocyte-associated protein 4 (CTLA-4) across multiple cancer types (Fig 2A). The analysis conducted using the Tumor Immune Estimation Resource (TIMER) database demonstrated a significant correlation between RAMP1 gene expression and various immune cell populations infiltrating the TME. The level of immune cell infiltration in pan-cancer seemed to differ based on RAMP1 gene expression. For example, RAMP1 expression was positively correlated with the infiltration of dendritic cells (DC) in BLCA. Conversely, a negative correlation was noted between RAMP1 expression and the infiltration of neutrophil and macrophages in Sarcoma (SARC) (Fig 2B). The analysis of RAMP1 gene expression and immune scores, including the ESTIMATE Score, Immune Score, and Stromal Score, revealed significant associations. High RAMP1 expression was positively correlated with ESTIMATE Score, Immune Score, and Stromal Score in colon adenocarcinoma (COAD), BLCA and READ (r>0.3, *p*<0.05) (Fig 2C–2E).

### Analysis of differential gene expression, prognostic significance, pathway enrichment, and protein-protein interaction (PPI) network construction in the GSE39058 dataset

**Fig 3:**
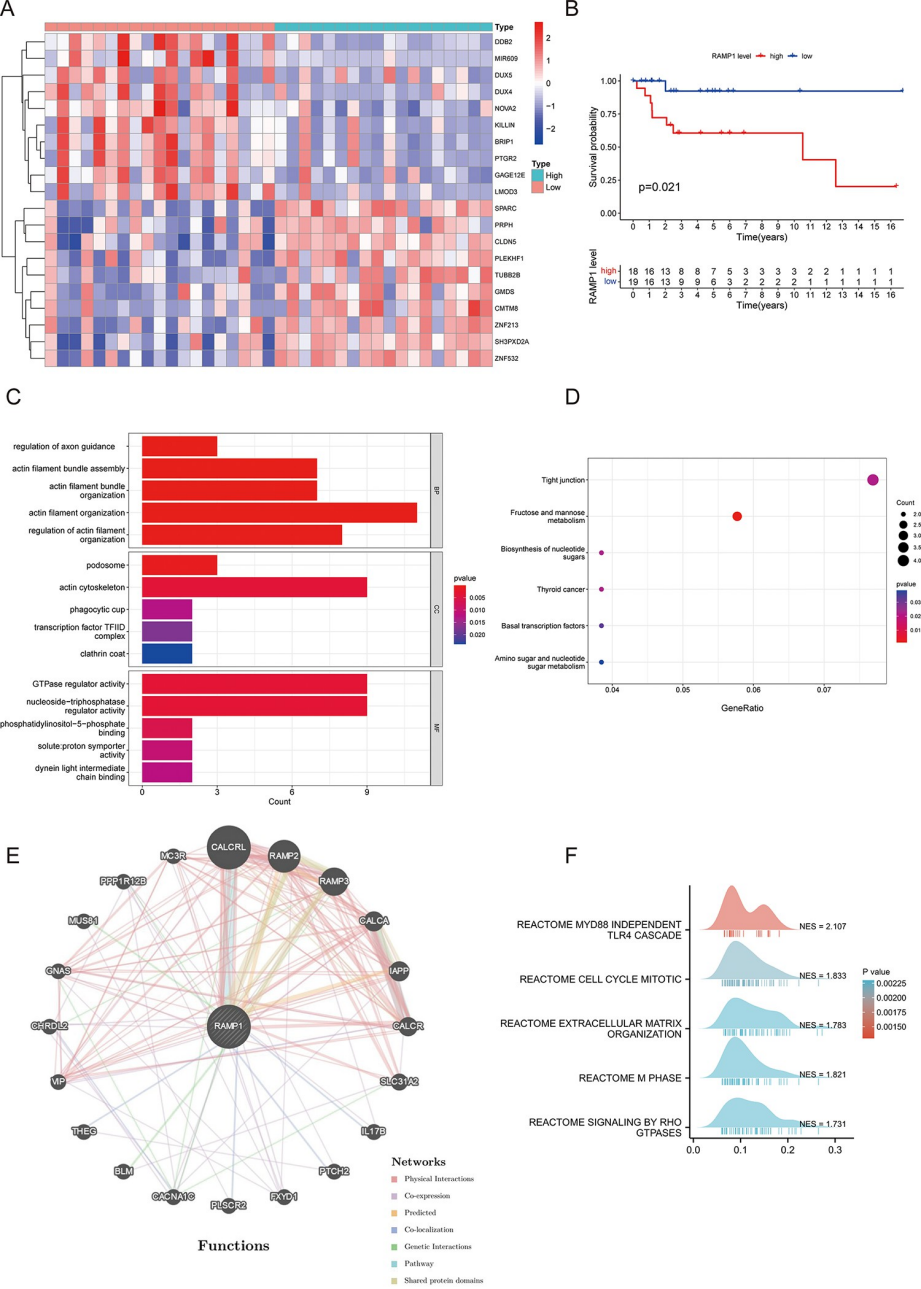
Prognostic value of RAMP1 in the GSE39058 dataset and Gene Ontology (GO) analysis. A heatmap of differentially expressed genes (DEGs) showing the top 10 upregulated and downregulated genes (3A). A higher RAMP1 gene expression is substantially related to a poorer prognosis in the GSE39058 dataset (*p* = 0.021) (3B). GO and Kyoto Encyclopedia of Genes and Genomes (KEGG) analyses were performed to determine the GO terms and pathways (3C) and KEGG terms (3D) associated with the DEGs. A protein-protein interaction (PPI) network of RAMP1 was generated using GeneMANIA (3E). Gene Set Enrichment Analysis (GSEA) identified significantly enriched signaling pathways (3F).

A heatmap in Fig 3A displays the top 10 up-regulated and down-regulated genes. Notably, a higher RAMP1 gene expression was significantly associated with poor prognosis in the GSE39058 dataset (*p* = 0.021) (Fig 3B). GO and KEGG analyses were performed to determine the GO terms and pathways related to the DEGs. Actin filament organization, actin-cytoskeleton, and GTPase regulator activity were the top GO terms discovered (Fig 3C). In addition, the top KEGG terms included tight junction and fructose and mannose metabolism (Fig 3D). Furthermore, a PPI network of RAMP1 was generated using GeneMANIA to investigate the potential relationships between co-expressed proteins (Fig 3E). GSEA plots indicated substantial enrichment of Reactome pathways such as MyD88-independent TLR4 cascade, mitotic cell cycle, extracellular matrix organization, M phase, and signaling by Rho GTPases (Fig 3F).

### Prognostic value of RAMP1

**Fig 4:**
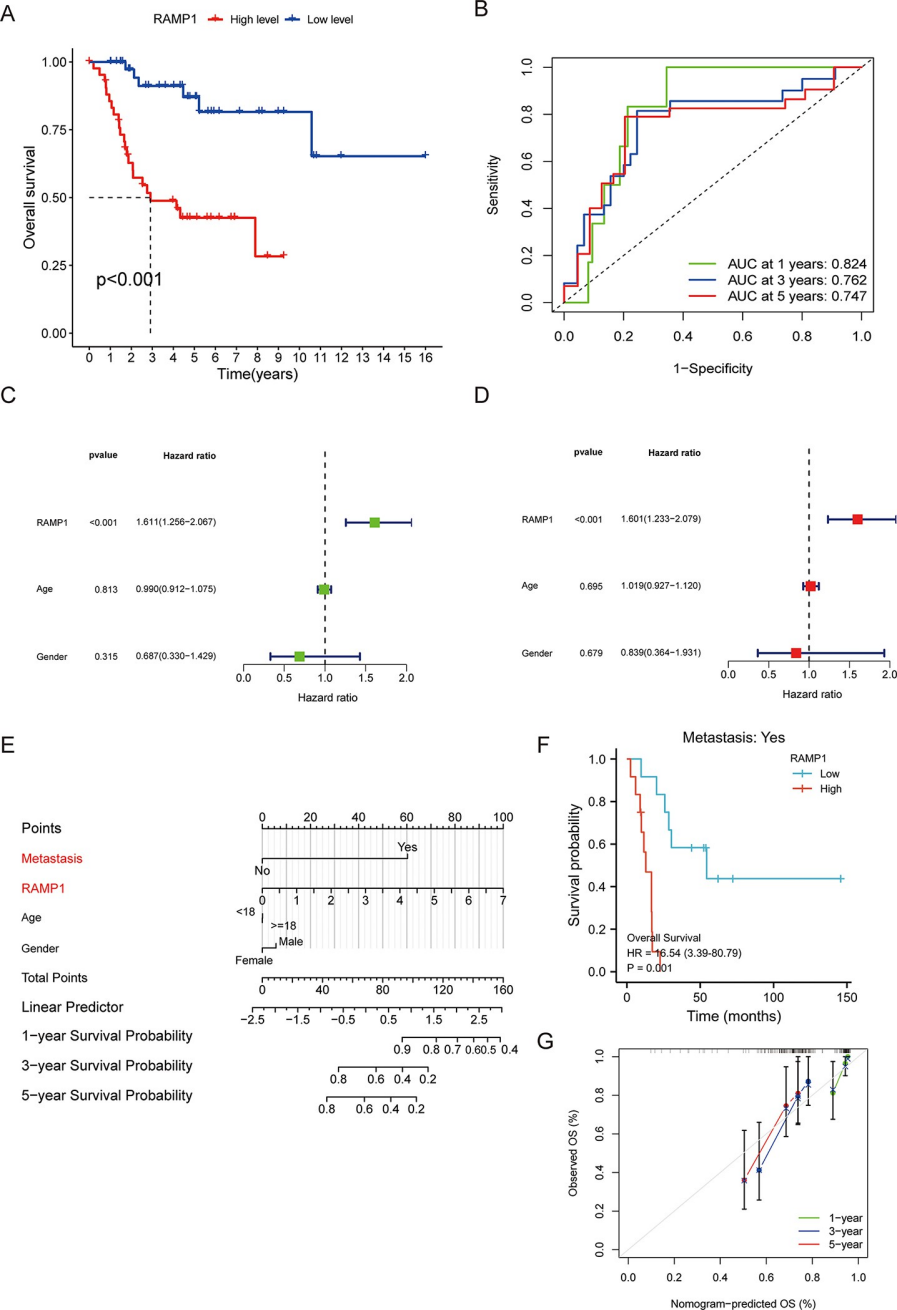
Prognostic value of RAMP1. High expression of RAMP1 correlates with poor prognosis (*p* < 0.001) (4A). The AUC value for 1‐, 3‐, and 5‐year survival were 0.824, 0.762, and 0.747, respectively (4B). The univariate (95% confidence interval [CI]  =  1.256–2.067, *p* < 0.001) (4C) and multivariable (95% CI  =  1.233–2.079, *p* < 0.001) (4D) analyses showed that RAMP1 was a significant independent prognostic risk factor. A nomogram (4E) and the ***calibration*** curve of the nomogram (4G) were constructed to predict the 1-, 3-, and 5-year survival of each patient. The prognosis of OS patients with metastasis was substantially unfavorable in the group with high RAMP1 gene expression compared to the group with low expression (4F).

High RAMP1 expression correlated with poor prognosis (*p* < 0.001) (Fig 4A). To assess the diagnostic ability of RAMP1 in OS, ROC analysis was performed and the results were summarized using the AUC scores. The 1‐, 3‐, and 5‐year survival analyses yield AUC values of 0.824, 0.762, and 0.747, respectively (Fig 4B). Furthermore, this study identified RAMP1 as a significant independent prognostic risk factor in both the univariate (HR  =  1.611, 95% CI  =  1.256–2.067, *p* < 0.001) (Fig 4C) and multivariable (HR  =  1.601, 95% CI  =  1.233–2.079, *p* < 0.001) (Fig 4D) analyses. A nomogram was constructed to predict the 1-, 3- and 5-year survival of each patient (Fig 4E). The calibration curve of the nomogram (Fig 4G) revealed that the nomogram was accurately calibrated. Patients with relatively high RAMP1 expression had significantly poor survival rates than those in the low-expression group (Fig 4F). in addition, the relationships between RAMP1 and several clinical features in OS were demonstrated and summarized in Table 1.

**Table 1:** Clinical characteristics of patients with OS.

Characteristic	levels	Low expression of RAMP1	High expression of RAMP1	*p*
n		50	51	
Metastasis n (%)	No	40 (52.6%)	36 (47.4%)	0.387
	Yes	10 (40%)	15 (60%)	0.001
Tumor region n (%)	Distal	18 (50%)	18 (50%)	0.917
	Other	2 (66.7%)	1 (33.3%)	
	Proximal	14 (56%)	11 (44%)	
	Proximal & Distal	0 (%)	0 (%)	
Age n (%)	<18	38 (48.7%)	40 (51.3%)	0.957
	> = 18	12 (52.2%)	11 (47.8%)	
Gender n (%)	Female	22 (53.7%)	19 (46.3%)	0.626
	Male	28 (46.7%)	32 (53.3%)	
OS event n (%)	Alive	38 (65.5%)	20 (34.5%)	<0.001
	Dead	11 (26.8%)	30 (73.2%)	
PFS event n (%)	No	31 (67.4%)	15 (32.6%)	0.002
	Yes	18 (34%)	35 (66%)	
Age, median (IQR)		15.08 (12.42, 17.64)	15.35 (12.35, 17.77)	0.978

### Analysis of differential gene expression, GO, KEGG, and GSEA in the TARGET dataset

**Fig 5:**
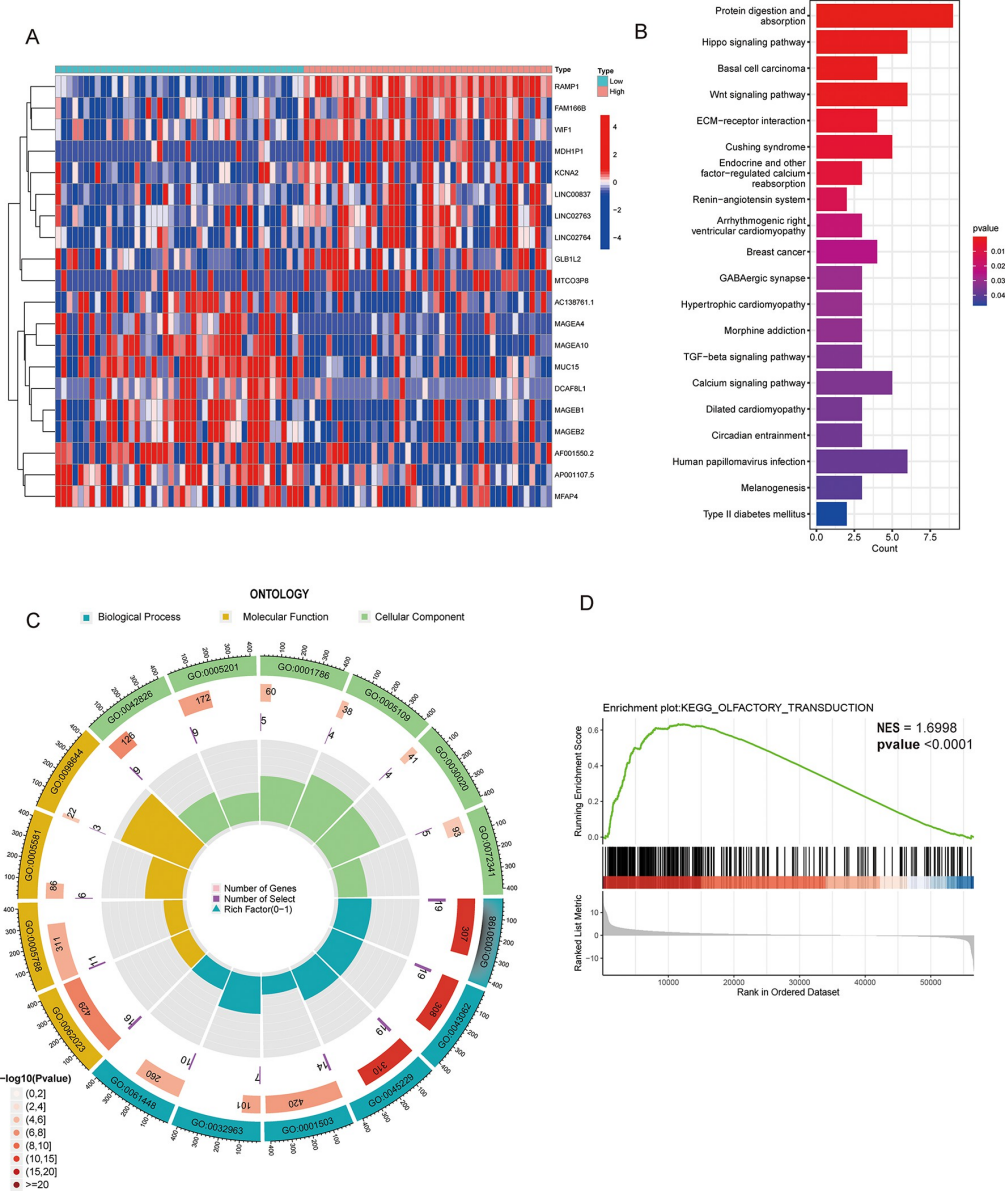
Differential gene expression, GO, KEGG, and GSEA analyses in Therapeutically Applicable Research to Generate (TARGET). A heatmap depicting the top 10 upregulated and downregulated genes (5A). The KEGG analysis showed that protein digestion and absorption, the Hippo signaling pathway, and the Wnt signaling pathway were significantly enriched (5B). Genes involved significantly in biological process (BP): extracellular matrix organization (GO:0030198), molecular function (MF): histone deacetylase binding (GO:0042826), and cellular component (CC): collagen-containing extracellular matrix (GO:0062023) are shown in a circle graph (5C). The GSEA analysis revealed that these DEGs were predominantly enriched in olfactory transduction pathways (normalized enrichment scores [NES] = 1.6998, *p* < 0.0001) (5D).

Fig 5A depicts a heatmap of the top 10 upregulated and downregulated genes. The KEGG pathway enrichment analysis revealed significant enrichment of protein digestion and absorption, Hippo signaling pathway, and the Wnt signaling pathway (Fig 5B). Genes involved significantly in biological process (BP): extracellular matrix organization (GO:0030198), molecular function (MF): histone deacetylase binding (GO:0042826), and cellular component (CC): collagen-containing extracellular matrix (GO:0062023) are shown in a circle graph in Fig 5C. The GSEA analysis revealed that these DEGs were predominantly enriched in olfactory transduction pathways (normalized enrichment score [NES] = 1.6998, *p* < 0.0001) (Fig 5D).

### Correlations between RAMP1 and its co-expressed genes

**Fig 6:**
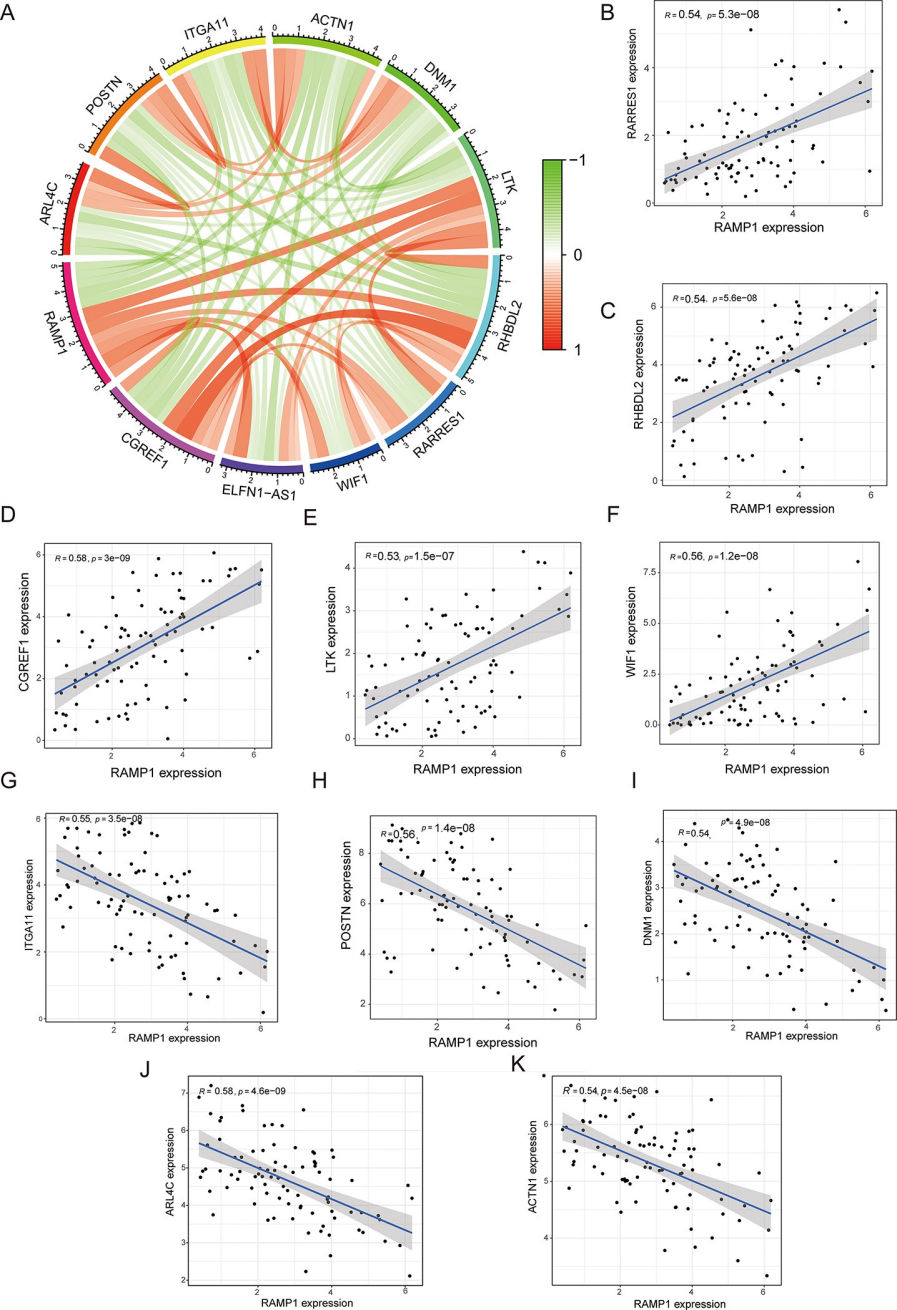
Correlation between RAMP1 and its co-expressed genes. The top five co-expressed genes that were upregulated or downregulated by the RAMP1 gene were identified using Pearson correlation analysis (6A). Upregulation and downregulation are shown by the red and green curves, respectively. The top five upregulated genes (CGREF1, LTK, RARRES1, RHBDL2, and WIF1) (6B - 6F) and downregulated genes (ACTN1, ARL4C, ITGA11, DNM1, and POSTN) (6G - 6K).

Fig 6A depicts the top five co-expressed genes that were upregulated or downregulated by the RAMP1 gene. The red and green curves represent upregulation and downregulation, respectively. The top five up-regulated genes (CGREF1, LTK, RARRES1, RHBDL2, and WIF1) and down-regulated genes (ACTN1, ARL4C, ITGA11, DNM1, and POSTN) are presented in Fig 6B–6K.

### Correlations between RAMP1 expression and tumor-infiltrating immune cells in OS

**Fig 7:**
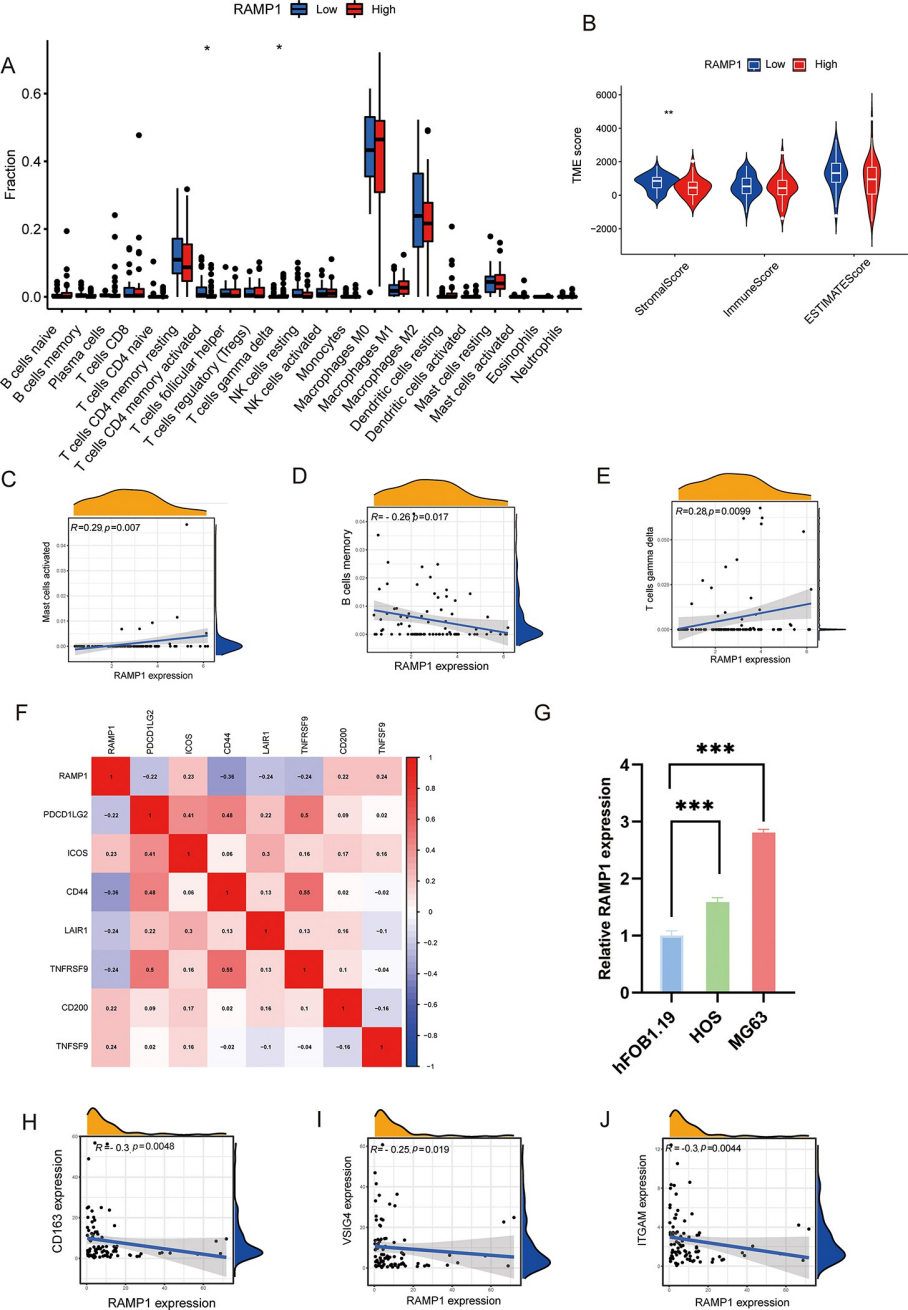
Correlation between RAMP1 expression and tumor-infiltrating immune cells. CD4+ memory-activated T cells and gamma-delta T cells varied considerably between the high and low RAMP1 expression groups (7A). Gamma-delta T cells, activated mast cells, and RAMP1 RNA levels were positively correlated (r > 0, *p* < 0.001) (7B and 7C), whereas memory B cells were negatively correlated with RAMP1 mRNA levels (r = -0.26, *p* = 0.017) (7D). The low RAMP1 expression group had significantly higher stromal scores than the high RAMP1 expression group (**p < 0.01) (7E). The correlation between the RAMP1 gene and immune checkpoint genes was presented (7F). As determined by real-time RT-PCR, the expression of the RAMP1 gene was higher in OS cell lines than in hFOB1.19 osteoblast cell lines (7G). RAMP1 expression was significantly associated with immunological markers in several immune cells, including CD163, VSIG4 (M2 macrophage), and ITGAM (neutrophil) (*p* < 0.05) (7H - 7J).

Fig 7A presents significant differences in the abundance of both CD4+ memory-activated T cells and gamma-delta T cells differed the high and low RAMP1 groups. Low RAMP1 expression was associated with high level of CD4+ memory-activated T cells. whereas a large proportion of gamma-delta T cells correlated with high RAMP1 expression levels. Furthermore, our investigation revealed a significant positive correlation between immune cell infiltration levels, including gamma-delta T cells and activated mast cells, and RAMP1 RNA levels (r > 0, *p* < 0.001) (Fig 7B and 7C). Moreover, there was a negative connection between memory B cells and RAMP1 expression (r = -0.26, *p* = 0.017) (Fig 7D). The association between RAMP1 expression and stromal score in the TARGET database is shown by a violin plot. The red violins represent the high RAMP1 expression group, whereas the blue violins represent the low RAMP1 expression group. There were no significant differences between the groups in terms of the immune score and ESTIMATE score. However, the stromal score of the low RAMP1 expression group was substantially higher than that of the high RAMP1 expression group (**p < 0.01) (Fig 7E).

### Correlation between RAMP1 expression, immune checkpoint genes, and immune cell markers, and validation of RAMP1 gene expression by quantitative reverse transcription-PCR (qRT-PCR)

The degree of correlation between the RAMP1 gene and immune checkpoint genes was examined using the "corrplot" package in the R software, and the results are shown in Fig 7F. The correlation matrix plots revealed an inverse relationship between RAMP1 expression and CD44 expression (r = -0.36, *p* < 0.05) and a positive correlation between RAMP1 expression and TNFSF9 expression (r = 0.23, *p* < 0.05). Moreover, RAMP1 expression was substantially linked with immunological markers in various immune cells, including CD163, VSIG4 (M2 macrophage), and ITGAM (neutrophil) (*p* < 0.05) (Fig 7H–7J). Additionally, qRT-PCR reveled elevated RAMP1 gene expression in OS cell lines compared to the osteoblast cell line hFOB1.19 (Fig 7G).

## Discussion

RAMP1, a mature glycoprotein receptor on the cell surface, complexes with calcitonin receptor-like receptor (CLR) to produce a high-affinity receptor for calcitonin-gene-related peptide (CGRP) receptor [20]. Since the first Food and Drug Administration (FDA) approval of G-protein-coupled receptor (GPCR)-directed antibody against RAMP1-CLR for the treatment of migraine in 2018 [21], the involvement of the RAMP1-CLR signaling axis in peripheral neurons has gained significant attention in recent years. A study reported that the CGRP-RAMP1 axis plays a crucial role in mucus production by goblet cells, which is mediated by sensory neurons and enhances the intestinal barrier [22]. In addition, researchers have used single-cell RNA sequencing analysis to demonstrate RAMP1 overexpression is associated with a poor clinical outcome in patients with melanoma [23]. Although RAMP1 has not been extensively explored, a growing body of literature shows that RAMP1 research may be the latest potential "hotspot" in disease development. Thus, in the current investigation, we sought to determine the potential value of RAMP1 in molecular diagnostics to provide an accurate prognosis for patients with OS. Notably, a series of bioinformatics analyses, coupled with in vitro experiments, suggested that RAMP1 could potentially serve as a novel therapeutic target in OS.

In the present study, high RAMP1 expression was associated with a poor prognosis in patients with OS as observed in the TARGET and GEO datasets. Additionally, the high expression of RAMP1 was found to have contributed to the metastatic phenotype based on the TARGET database. Both univariate and multivariate analyses indicated that high RAMP1 expression is an independent predictive factor for patient survival in OS. Furthermore, the ROC analysis revealed that the AUC values for RAMP1 at 1, 3, and 5 years were all higher than 0.70, further supporting the potential of RAMP1 expression as a prognostic factor in OS. Our findings support recent research, suggesting that RAMP1 functions as a prognostic biomarker. This implies that it holds promise in predicting patient outcomes and informing treatment strategies for osteosarcoma [24]. Moreover, the GSEA analysis of signaling pathways revealed significant enrichment of RAMP1 in the olfactory transduction processes, providing insight into the mechanisms underlying its prognostic role. Consistent with the current conclusion, earlier research has indicated that olfactory transduction processes may be a potential mechanism associated with OS prognosis [25]. In addition, the KEGG pathway analysis revealed an abundance of genes involved in the Hippo and Wnt signaling pathway. Consistent with our findings, previous research has shown the significance of the Hippo and Wnt signaling pathway in OS. indicating that these two signaling pathways might be potential therapeutic targets in OS [26, 27].

Additionally, considering the crucial role of immune cell infiltration in the tumor-immune microenvironment during the progression of OS [28], we further explored the association between the intra-tumoral immune microenvironment and RAMP1 in OS. Notably, we observed significant differences in the infiltration of two immune cell types, specifically T cells CD4 memory activated and T cells gamma delta (γδ T Cells), between patients with OS who had high and low RAMP1 expression. Previous research has shown that the infiltration of activated memory CD4 T -cells is associated with distinct prognostic outcomes in OS and may function as specific immune targets. Similarly, the therapeutic potential of ex vivo expanded γδ T cells against OS cells was also observed, indicating that γδ T cells have the potential to enhance efficacy of chemotherapeutic drugs against OS and may represent a novel immunotherapy method [29]. Moreover, the expression level of CD163, a surface marker of M2 macrophages, was inversely correlated with the expression level of RAMP1, Higher RAMP1 expression was strongly associated with poor prognosis. We hypothesized that a high level of CD163 was related to a favorable outcome in patients with OS. Consistent with our findings, earlier research has shown a significant association between high CD163 levels and a higher overall survival rate in patients with OS [30].

We acknowledge that our research also has limitations. First, although we provided bioinformatics evidence and validated the mRNA expression of RAMP1 in vivo, we did not present in vitro and in vivo evidence for the biological mechanism of RAMP1 in OS, nor did we compare its abundance in OS tissue to normal tissues. Second, our research had a limited sample size derived from public data sets and lacked a validation cohort obtained from our data collection.

In summary, the current research demonstrates the diagnostic, prognostic, and potential therapeutic value of RAMP1 in OS, presenting it as a novel therapeutic target.

## Conclusion

In conclusion, our study provides initial insights into the potential role of RAMP1 in patients with OS. Our findings suggest a significant association between RAMP1 and the metastases and prognosis of OS. Specifically, patients with OS who had high RAMP1 expression exhibited a relatively poorer prognosis. These results highlight the potential of RAMP1 as a prognostic biomarker and molecular therapeutic target for OS.
